# Supplementary material for: Programmed biomolecule delivery to enable and direct cell migration for connective tissue repair
Source: Nat Commun. 2017 Nov 24;8:1780. doi: 10.1038/s41467-017-01955-w (PMC5701126; doi:10.1038/s41467-017-01955-w)
Supplement: Supplementary file 1 — Supplementary Information [file 41467_2017_1955_MOESM1_ESM.pdf]

a

Polymer	Flow rate (mL/hr)	Concentration (g/mL)	Flow rate (g/hr)	Theoretical Fiber Fraction (% mass)
PCL	2	0.143	0.286	50.2
PEO	2.5	0.08	0.2	35.1
HA	1.4	0.06	0.084	14.7

b

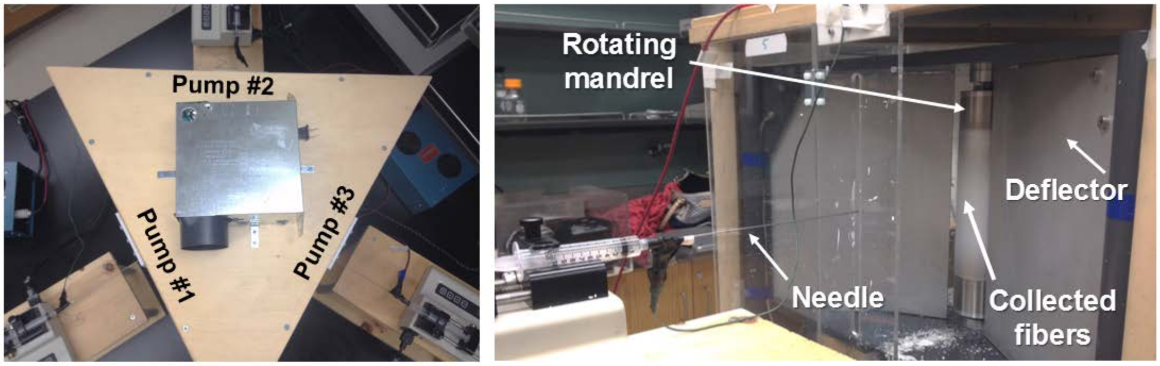

Supplementary Figure 1: Fabrication of tri-component nanofibrous composite scaffolds. (**a**) Polymer solution parameters and fiber fraction calculations (% mass) based on solution flow rate and concentrations. (**b**) Images of tri-jet electrospinning setup.

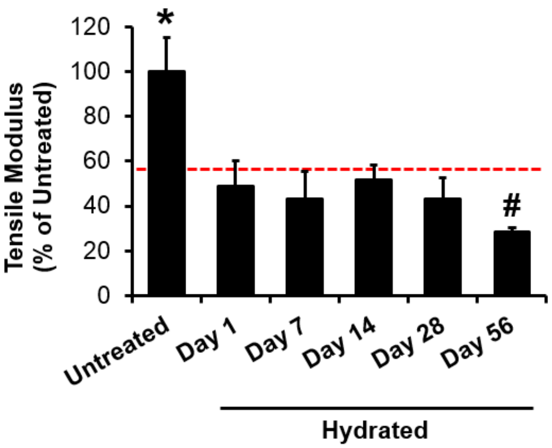

Supplementary Figure 2: Scaffold mechanics is dictated by the PCL fiber fraction. Tensile modulus of PCL/PEO/HA scaffolds before (Untreated) and after various periods of hydration (Hydrated), shown as a percentage of the Untreated condition (*n*=4–6 samples/group, mean ± s.d.). Red dashed line indicates average value for scaffolds treated with hyaluronidase (HASE) for 24 hours. *=p<0.05 vs. all other groups, #=p<0.05 vs. HASE treatment.

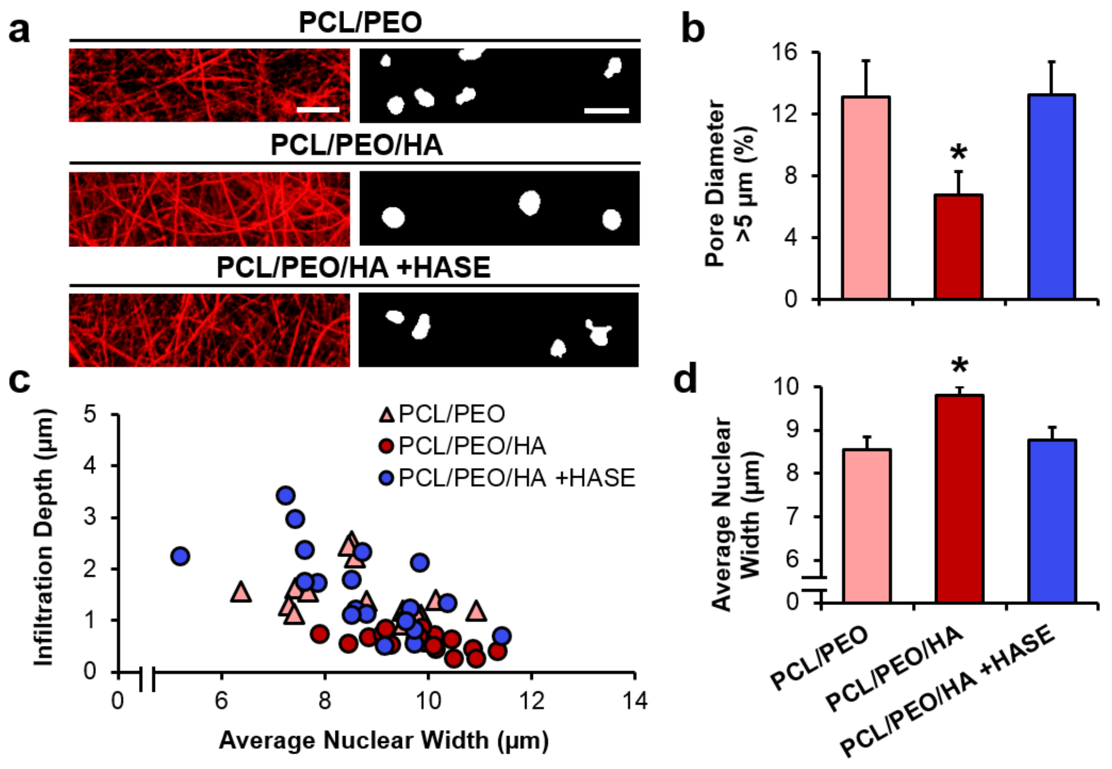

Supplementary Figure 3: Pore size and nuclear deformation during migration through nanofibrous scaffolds. (**a**) Top-down projections of rhodamine-doped fibers (left) and DAPI-stained cell nuclei (binarized signal). Scale=20 µm. (**b**) Population (%) of pores >5 µm in diameter (*n*=3 samples/group, mean ± s.d.). (**c**) Infiltration depth as a function of average nuclear width (*n*=20 cells/group). (**d**) Average nuclear width (*n*=20 cells/group, mean ± s.e.m.). *=p<0.05 vs. all other groups.

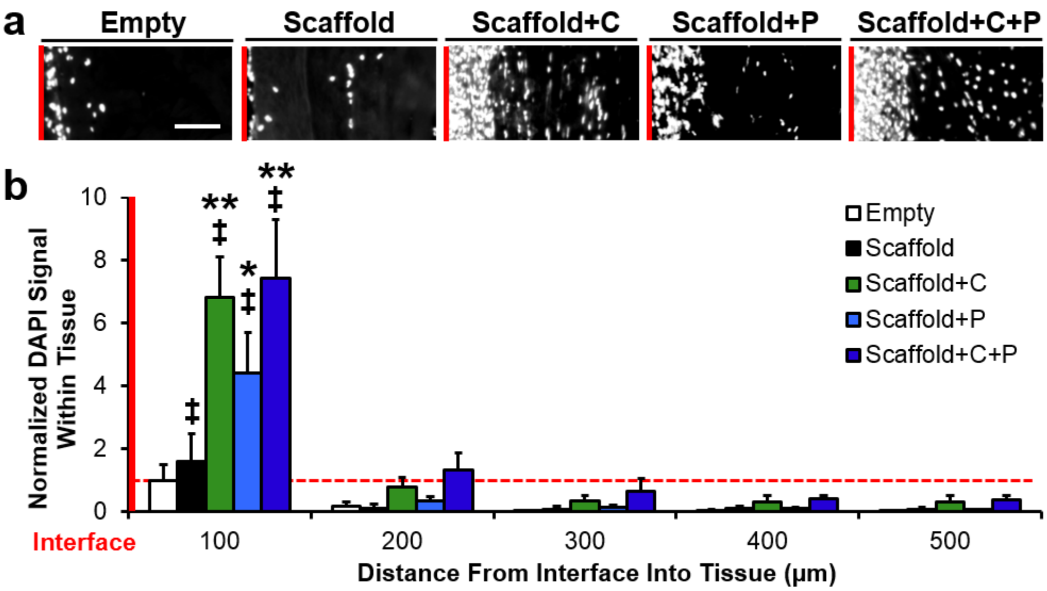

Supplementary Figure 4: Scaffolds releasing collagenase and PDGF-AB increase interfacial cellularity after 2 weeks of subcutaneous implantation. (**a**) DAPI staining showing nuclei at the interface (red line). Scale=100 µm. (**b**) Average intensity with respect to distance from the interface, normalized to the Empty value at the interface (n=4–5 samples/group, mean ± s.d.). ‡=p<0.05 vs. all other distances, *=p<0.05 vs. Empty and Scaffold, **=p<0.05 vs. Empty, Scaffold, and Scaffold+P.

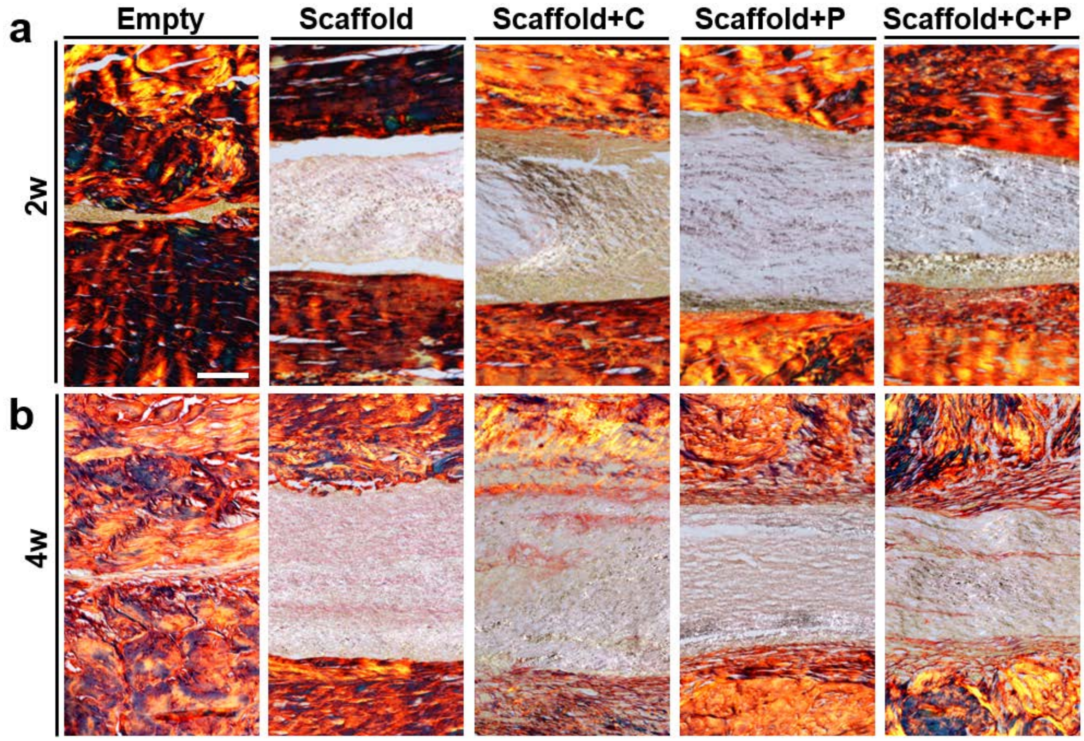

Supplementary Figure 5: Bioactive scaffolds increase interfacial matrix remodeling over time. (**a**) Picrosirius Red staining of repair constructs showing collagen and nanofibrous scaffolds at the wound site at 2 weeks and (**b**) 4 weeks after subcutaneous implantation. Scale=100 µm.

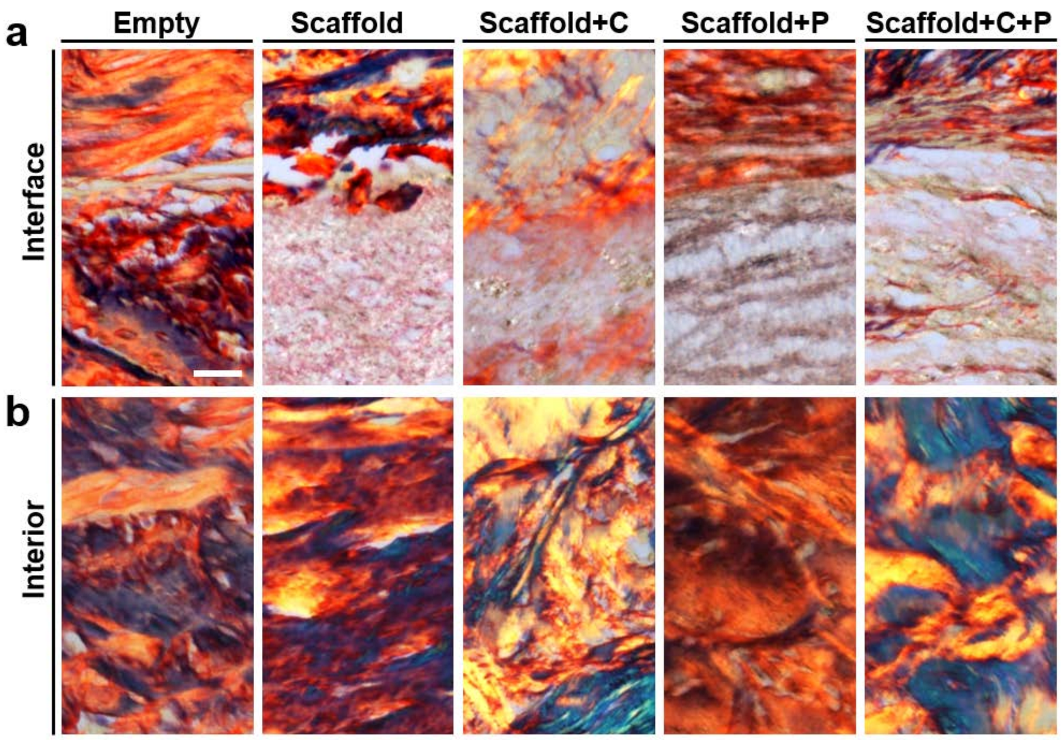

Supplementary Figure 6: Matrix degradation is localized to the wound interface. (**a**) Zoomed in images showing Picrosirius Red staining of repair constructs at the wound site and (**b**) within the explant interior (300 µm away from the wound site) at 4 weeks after subcutaneous implantation. Scale=20 µm.

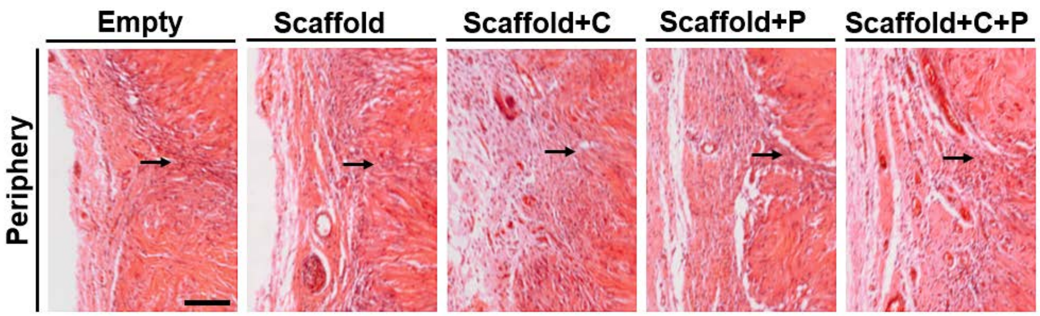

Supplementary Figure 7: Host response at the explant periphery is minimal. H&E staining of repair construct boundaries at 4 weeks after subcutaneous implantation, where arrows indicate the wound interface at the explant edge. Fibrous tissue capsule formation and evidence of new blood vessels are observed at the explant periphery in all groups. Scale=100 µm.
